# The Association of Body Image Perceptions with Behavioral and Health Outcomes among Young Adults

**DOI:** 10.3390/nu16091281

**Published:** 2024-04-25

**Authors:** Jorge Jiménez-Morcillo, Domingo Jesús Ramos-Campo, Stephanie Rodríguez-Besteiro, Vicente Javier Clemente-Suárez

**Affiliations:** 1 Faculty of Sports Sciences, Universidad Europea de Madrid, Tajo Street, s/n, 28670 Madrid, Spain; jorge.jimenez2@universidadeuropea.es (J.J.-M.); annie310791@gmail.com (S.R.-B.); vctxente@yahoo.es (V.J.C.-S.); 2 LFE Research Group, Department of Health and Human Performance, Faculty of Physical Activity and Sport Science (INEF), Universidad Politécnica de Madrid, 28040 Madrid, Spain; 3 Grupo de Investigación en Cultura, Educación y Sociedad, Universidad de la Costa, Barranquilla 080002, Colombia

**Keywords:** body image perception, dietary habits, physical activity, strength training, mental health, lifestyle choices, self-perception

## Abstract

This study was conducted on 5 March 2024, by the Universidad Europea de Madrid. This study aims to explore how body image perceptions influence health behaviors and mental and physical health outcomes among a specific group of 605 young adults aged 20 to 35 engaged in strength training regimes. To measure these perceptions, the Multidimensional Body-Self Relations Questionnaire (MBSRQ) was employed, an advanced tool that assesses multiple dimensions of body image through its subscales, including feelings of physical attractiveness, investment in one’s appearance, and satisfaction with different body areas. Participants were segmented into two groups based on a median split of their self-reported body image. The survey assessed a diverse array of variables, including demographic details, physiological data, resistance training routines, and psychological attributes. In this revision, we consistently use the term ‘dietary habits’ to ensure clear and uniform language when discussing eating patterns. Notable differences were observed in dietary habits and exercise engagement, influenced significantly by body image perceptions. Negative body image was linked to less healthy dietary habits, diminished physical activity, and worse psychological outcomes, such as increased anxiety and depression. Conversely, a positive body image was associated with healthier dietary habits, more frequent physical activity, and better psychological health. The findings suggest that interventions aimed at improving body image could be crucial for enhancing overall health within this specific demographic. Due to the non-representative nature of the study group, conclusions are cautiously presented as applicable only to similar populations engaged in strength training. This study underscores the need for holistic strategies that encourage positive body image to improve both physical and psychological health outcomes in young adults.

## 1. Introduction

Body image is a multifaceted construct that encompasses an individual’s perceptions, emotions, and cognitions about their own body, influenced by personal experiences, sociocultural norms, and media portrayals [1,2]. It acts as a crucial interface between internal self and external societal pressures, deeply shaping self-perception and self-worth. The importance of understanding body image’s role in influencing mental and physical health is increasingly recognized, especially as it significantly affects self-esteem and overall well-being [3]. Recent increases in body image concerns among adolescents and young adults call for targeted research to develop interventions that address the complex dynamics of body image perception and its negative impacts, including eating and depressive disorders [4,5]. This period of transition is marked by heightened vulnerability to societal and peer influences, which can exacerbate body image dissatisfaction and lead to long-term health issues [6]. The ongoing redefinition of societal ideals, amplified by digital media, underscores the need for a comprehensive approach to mitigate these concerns and promote a healthier societal view of body image [7,8,9].

This study focuses on body image due to its significant influence on self-esteem and overall well-being, reflecting a complex amalgamation of personal experiences, sociocultural norms, and media representations. The importance of investigating body image lies in its ability to impact health-related behaviors, decisions, and psychological well-being, especially during the critical transition from adolescence to young adulthood—a period marked by vulnerabilities to external influences and substantial physical, emotional, and social changes. By exploring how body image perceptions correlate with healthy or detrimental behavioral patterns, this study aims to develop effective interventions that promote a positive body image and, consequently, better well-being among young adults, addressing an urgent need highlighted by the increasing prevalence of body image concerns. This targeted approach allows for precise research, avoiding the indiscriminate exploration of variables without prior hypotheses, and ensures that each examined element significantly contributes to our understanding of body image and its extensive associations with mental and physical health.

The distortion of body image has a profound and multifaceted association, affecting psychological well-being, nutritional behaviors, and social relationships [10]. Psychologically, distorted body image perceptions can lead to severe mental health issues, including anxiety, depression, and body dysmorphic disorders [11]. Nutritionally, these distorted perceptions may result in unhealthy eating patterns, ranging from restrictive dieting to binge eating, significantly affecting individuals’ physical health [12]. Socially, the association extends to relationships and social interactions, where dissatisfaction with body image can diminish self-confidence, hinder social engagement, and affect the quality of interpersonal relationships [13]. This study aims to explore the relationship between body image perceptions and their consequential effects on behavioral patterns, health-related decisions, and psychological well-being among young adults. We hypothesize that negative body image perceptions will correlate with unhealthy behaviors, such as poor eating habits and physical inactivity, and adverse mental health outcomes, including anxiety and depression. Conversely, positive body image perceptions are expected to be associated with healthier behavioral patterns, better psychological resilience, and improved overall well-being, thereby highlighting the critical need for interventions aimed at improving body image perceptions among young adults.

## 2. Materials and Methods

In our study, conducted on 5 March 2024, at the Universidad Europea de Madrid, we surveyed 605 individuals involved in strength training via an online questionnaire, including 385 males and 224 females. Participants were recruited through a dedicated Google Forms link or by scanning a QR code, both of which were widely distributed across various online platforms and at the university campus to ensure a broad and diverse representation of the young adult population engaged in strength training. All participants in the study adhered to a type of healthy yet disorganized diet and did not exhibit any health issues. Both parameters were used as exclusion criteria for the study. Before their participation, individuals were comprehensively informed about the objectives and methodology of the research. The inclusion criteria required participants of both genders to be between the ages of 20 and 35 and to have engaged in strength training activities 2 to 7 days per week for a minimum duration of 6 months. We rigorously reviewed each response for precision and completeness to guarantee the reliability of the data. An initial assessment was conducted to identify any potential biases or anomalies in the responses. Participation was entirely voluntary, with participants having the freedom to withdraw at any point. Digital consent was obtained through a signed informed consent form, verifying their understanding and agreement to participate in the study, adhering to the ethical standards outlined in the Helsinki Declaration (revised in Brazil, 2013) regarding ethical research involving human subjects. The University Ethics Committee (CIPI/21/082) approved the study, ensuring compliance with ethical guidelines. To measure body image perceptions, validated psychometric scales such as the Body Image Assessment (BIA) and the Multidimensional Body-Self Relations Questionnaire (MBSRQ) were employed. These instruments enabled quantitative evaluations of body image dimensions, ensuring robust and reproducible measurement techniques. To address potential confounding factors such as weight status that could influence the outcomes of our study, we included weight as a covariate in our statistical analysis. This adjustment allowed us to discern the specific impacts of body image perception (BIP) on health and behavioral outcomes, independent of any weight-related effects. This methodological refinement ensures that the associations we report between BIP and various outcomes are robust and not unduly influenced by participants’ weight status.

In this study, a correlational approach was adopted to examine the relationships between body image perceptions and a range of health and behavioral outcomes in young adults. It is crucial to understand that, although we identified statistically significant correlations between these variables, the nature of our research design implies that conclusions regarding direct causality are not feasible. Statistical adjustments, including the Bonferroni correction, were performed to control the risk of Type I errors due to the number of multiple comparisons, ensuring the robustness of our correlational inferences.

In structuring the sample for our study, we categorized participants into two groups based on their body image perception, employing the 50th percentile as the division criterion. Individuals whose perception of their body image was at or below the median were classified into the lower body image perception group (LBIP), representing those with potentially more negative or critical views towards their own bodies. This segmentation offered critical insights into the association of less positive self-image, which is crucial for understanding the varied attitudes and feelings towards one’s body within the population. Conversely, participants with body image perceptions above the median were allocated to the higher body image perception group (HBIP), indicative of a cohort with more satisfied or positive perceptions of their body image within our study. This delineation allowed for an in-depth examination of the outcomes associated with different levels of body image perception. The bifurcation of these groups facilitated a nuanced comparison, employing a median split approach to ensure an equitable comparison between participants with lower and higher body image perceptions.

In our study, we assessed body satisfaction among strength-trained individuals using the Eating Disorder Inventory (EDI), which includes a numerical scale from 1 (never) to 6 (always) and a body silhouette scale from 1 (thinnest) to 9 (most voluminous). Data were analyzed using SPSS version 24.0, where we computed descriptive statistics and employed Kolmogorov–Smirnov tests to verify data normality and homogeneity. Differences in various psychosocial and demographic variables were further examined using independent t-tests with a significance threshold set at *p* ≤ 0.05.

In the analytical component of our study, psychological traits and health-related variables among participants were systematically assessed and compared across two groups, differentiated by their body image perception—the lower body image perception group (LBIP) and higher body image perception group (HBIP). We utilized established psychological scales such as the Big Five Inventory to measure traits like extraversion, pleasantness, scrupulousness, neuroticism, and openness to experience. Additionally, mental health states were quantified using the Zung Self-Rating Depression Scale, the Acceptance and Action Questionnaire II (AAQ.ii), the UCLA Loneliness Scale, and the Spielberger State-Trait Anxiety Inventory (STAI). Physical health indicators, including the frequency of certain health symptoms like gastritis, dry throat, and dental sensitivity, were also recorded. Statistical differences between the LBIP and HBIP groups were analyzed using independent *t*-tests, providing a comprehensive view of the nuanced relationships between body image perceptions and a range of psychological and physical health outcomes.

In the analysis of our dataset, to account for the multiple comparisons inherent in assessing a broad array of variables—spanning demographic, physiological, dietary, psychological dimensions, and body satisfaction metrics—a Bonferroni correction was applied. Given the extensive range of comparisons, totaling 31 distinct analyses, the standard alpha level of 0.05 was adjusted to a more stringent threshold of approximately 0.0016. This adjustment ensures a conservative approach to statistical significance, thereby mitigating the potential for Type I errors and enhancing the reliability of our findings.

**The variables studied included the following:**

*Demographic and Physiological Metrics*: We recorded essential demographic and body metrics such as gender, age, height, weight, and Body Mass Index (BMI).

*Resistance Training experience*: The participants detailed their weekly exercise routines, encompassing aerobic and strength training sessions. The duration of aerobic exercises was measured in minutes per week, whereas the intensity of strength training was categorized by the percentages of weekly training relative to their one-repetition maximum (1RM): under 50%, between 50% and 70%, between 70% and 85%, and above 85%. Additionally, the percentages of 1RM for exercises like the back squat, deadlift, and bench press were recorded.

*Dietary Consumption Patterns*: Through a food frequency questionnaire, we evaluated the consumption of various food and beverage items, including juices (250 mL), water (250 mL per glass), alcoholic and fermented beverages (250 mL), soft drinks (250 mL), energy drinks (250 mL), milk (250 mL per glass), fruits (90 g), bakery/sweets (90 g), meat (150 g), fish (150 g), legumes (200 g), pasta or rice (150 g), vegetables (200 g), bread (50 g), fast food (180 g), whole foods (150 g), gel (40 g), muesli bars (150 g), and protein drinks (300 mL), based on methodologies from prior studies [14,15].

*Eating Behaviors*: Information regarding the frequency of consuming takeout food, dining out, and eating home-prepared meals was compiled.

*Psychological Dimensions*: A variety of scales and assessments were utilized to evaluate psychological attributes. Personality traits were measured using the Big Five Inventory in its Spanish version (alpha coefficient of 0.73) [16]. Anxiety was assessed with the Spanish adaptation of the Spielberger State-Trait Anxiety Inventory (alpha coefficient of 0.93) [17]. Experiential avoidance or psychological inflexibility was evaluated by the Spanish version of the Acceptance and Action Questionnaire II (alpha coefficient of 0.84) [18]. Feelings of loneliness were measured with the Spanish adaptation of the UCLA Loneliness Scale (alpha coefficient of 0.94) [19]. Depression levels were determined using the Spanish adapted version of the Zung Depression Scale for the COVID-19 context, which had an alpha coefficient of 0.09, with sensitivity and specificity both above 80% [20].

*Body Satisfaction Assessment*: The levels of body satisfaction among strength-trained individuals, differentiated by gender, were evaluated using the Eating Disorder Inventory [21]. This included a numeric scale ranging from 1 (never) to 6 (always) and a body silhouette scale from 1 (thinnest) to 9 (most voluminous).

*Data analysis*: Statistical analyses were conducted with SPSS version 24.0. We computed descriptive statistics and assessed the normality and homogeneity of the dataset using Kolmogorov–Smirnov tests. Differences in nutritional, sociodemographic, academic, and psychological aspects were examined using an independent *t*-test, with a significance threshold set at *p* ≤ 0.05.

## 3. Results

This section outlines the key findings from our study on the relationship between body image perception and its associated dietary, psychological, and physical activity patterns. Through a comparative analysis of individuals with low and high body image perception, we reveal notable differences across these domains, highlighting the breadth of body image’s association with lifestyle and health (Table 1).

Our assessment of dietary habits in relation to body image perception identified significant differences across a variety of food and beverage categories between individuals with low body image perception (LBIP) and those with high body image perception (HBIP). Differences in weekly consumption patterns were observed, including variations in the intake of energy drinks, milk, fermented dairy products, eggs, meat, fish, legumes, processed meats, whole foods, rice, pasta, bread, fruits, and vegetables. These findings illustrate diverse dietary preferences associated with body image perception, underscoring the potential for tailored nutritional guidance and interventions based on body image (Table 2).

Exploring the intersection of psychological traits, body image perceptions, and health symptoms, our study found that differences in body image perception were associated with variations in the Big Five personality traits—extraversion, agreeableness, conscientiousness, and openness to experience—as well as body satisfaction levels as measured by the Eating Disorder Inventory (EDI) Body Satisfaction scale. Additionally, the prevalence of health symptoms such as frequent gastritis or heartburn, dry throat, and dental sensitivity varied between the LBIP and HBIP groups, indicating a relationship between body image perception and certain health outcomes (Table 3).

Analysis of physical activity patterns and their intensity among individuals with differing body image perceptions revealed distinctions in engagement in weekly aerobic training and performance in strength-based exercises, including personal records (PRs) in the bench press and back squat. Differences were observed in the distribution of training intensities, with notable variations in the percentages of training time allocated to below fifty percent, between seventy and eighty-five percent, and above eighty-five percent of maximum load. Furthermore, individuals with higher body image perception (BIP) exhibited a greater consumption of energy drinks. They also demonstrated higher intakes of fruits and vegetables, which aligns with healthier eating patterns.

These patterns suggest an association between body image perception and approaches to physical training, highlighting the influence of body image on physical fitness and training strategies (Table 4).

## 4. Discussion

Our study provides a detailed examination of how body image perceptions influence behavioral patterns, health decisions, and psychological well-being among young adults engaged in strength training. It is essential to recognize that eating disorders and body image perceptions disproportionately affect women compared to men [22,23]. It is critical to recognize the correlational nature of these findings; significant associations identified between body image and various health and behavior indicators do not imply causation. The use of the Bonferroni correction for 31 comparisons highlights our rigorous statistical approach, helping to prevent false-positive results and strengthen the validity of our conclusions [23]. Our results confirm the hypothesized link between negative body image perceptions and unhealthy behaviors like poor dietary habits and reduced physical activity, as well as adverse psychological outcomes such as anxiety and depression. Conversely, positive body image perceptions are associated with healthier behaviors, better psychological resilience, and overall well-being [24,25,26]. This study not only supports the substantial impact of body image on personal health and activity patterns but also emphasizes the need for addressing body image issues through targeted mental and physical health interventions, especially for those at risk of negative self-perceptions [27,28].

Our comparative analysis revealed marked differences in dietary habits between individuals with low and high body image perceptions, highlighting the complex interplay between body image and nutritional choices. Those with lower body image perceptions typically consumed more traditional foods such as milk, eggs, meat, and whole foods, while individuals with higher perceptions favored energy drinks [29,30,31]. This pattern suggests that body image may influence eating behaviors in ways that reflect both psychological needs and health aspirations, contradicting some traditional assumptions about dietary choices and body image [32,33]. These findings underline the necessity for personalized nutritional advice that addresses not only dietary preferences but also the underlying psychological factors associated with body image. Such guidance could encourage healthier eating patterns based on quality rather than merely caloric content, particularly for those with distorted body image perceptions who might benefit from interventions focused on improving both food quality perception and overall dietary habits [34,35,36].

The analysis of differences in psychological characteristics between individuals with low and high body image perception sheds light on the profound influence that self-image can exert on the psyche and physical health. Those with a high perception of their body image exhibit greater extraversion, agreeableness, conscientiousness, and openness to new experiences, findings that resonate with previous studies linking a positive body image to constructive personality traits and healthy outcomes, both mentally and physically [37]. Conversely, individuals with a low perception of their body image demonstrate lower body satisfaction and an increased predisposition to health issues such as gastritis, dry throat, and dental sensitivity. These latter results reflect the evidence from the literature suggesting that body dissatisfaction may be associated with a variety of negative health consequences [38], including specific physical symptoms that could be interpreted as somatic manifestations of negative psychological or emotional stress [39]. The lower body satisfaction among individuals with negative perceptions of their body image underscores the importance of body image in psychological interventions, aligning with the literature that consistently relates body dissatisfaction with adverse mental health outcomes, such as a predisposition to eating disorders and depression [40]. Furthermore, the association between a low perception of body image and an increase in the incidence of physical symptoms suggests an interconnection between emotional and physical well-being, in line with previous studies exploring how emotional distress can manifest in physical symptoms through chronic stress or negative health behaviors [41,42]. On the other hand, the correlation between a high body image perception and positive personality traits highlights how satisfaction with one’s appearance can promote a more positive attitude towards life and social interactions, as suggested by other authors [43,44]. This relationship not only reflects but could also contribute to a healthy psychological profile, reinforcing the notion that promoting a positive body image could be an effective strategy for improving overall well-being and mental health, as suggested in the existing literature [45]. This discussion underscores the need for therapeutic approaches that consider body image not only as a component of mental health but also as a significant influencer on physical health and personality traits. Such findings are imperative for the development of multidisciplinary interventions aimed at promoting a positive body image perception, thereby fostering an improvement in overall well-being and health, as supported by previous research [46].

The analysis of the relationship between body image perception and physical activity patterns reveals findings that align with previous studies [47,48], highlighting how self-image can influence and be influenced by sports behavior. The results indicate that individuals with a low perception of their body image tend to devote more time to aerobic training and achieve higher personal records (PRs) in both bench press and back squat, corroborating the findings of other authors who link a negative body image with a greater obsession with exercise [49,50]. Additionally, the data show differences in the distribution of training according to intensity, with individuals with a low perception of their body image dedicating a larger proportion of their training to intensities below fifty percent of their maximum load. Conversely, those with a high perception of their body image seem to distribute their training more evenly among different intensities, including a significant percentage of their training above eighty-five percent of their maximum load, consistent with information provided by existing studies [51,52]. This pattern underscores greater confidence and willingness to face more demanding physical challenges among individuals more satisfied with their body appearance, in line with previous studies [53,54]. This link between body image perception and the focus on high-intensity training highlights the importance of considering psychological aspects when designing personalized training programs. Satisfaction with body image can motivate individuals to engage in regular physical exercise, contributing to an improvement in physical strength and overall well-being, corroborating the results of other authors [55,56]. The discussion of these findings suggests that promoting a positive body image may be a crucial component in motivating individuals towards achieving more ambitious physical goals, enhancing their health and general well-being, as indicated by previous authors [57,58,59].

This study is not without its limitations. Firstly, the reliance on self-reported data may introduce bias, as participants might underreport or overreport behaviors or perceptions. Secondly, the cross-sectional design limits our ability to infer causality between body image perceptions and health outcomes. Thirdly, our sample is confined to young adults engaged in strength training, which may not fully represent the broader population’s experiences with body image issues. Lastly, cultural, social, and environmental factors influencing body image perceptions were not extensively explored, suggesting a need for more diverse and inclusive research. Another limitation of our study that warrants discussion is the uneven ratio of males to females among participants, along with differences in BMI categories between the two groups. The predominance of males may have influenced the generalizability of our findings across genders. Additionally, variations in BMI categories between the groups could have impacted the association between body image perceptions and health outcomes. These factors are critical to consider when interpreting the study results, as they might affect the robustness and applicability of our findings to a broader population. Future studies should aim to balance gender participation and more closely match participants across different BMI categories to enhance the external validity of the research.

Future studies should aim to address the limitations mentioned by adopting longitudinal designs to better understand the causal relationships between body image perceptions and health behaviors. Research should also expand beyond strength training enthusiasts to include a broader demographic, exploring how body image perceptions vary across different populations, including varying age groups, cultural backgrounds, and levels of physical activity. Additionally, future work should explore the effectiveness of targeted interventions designed to improve body image perceptions and examine their long-term associations on health behaviors and outcomes. Investigating the role of digital and social media in shaping body image perceptions remains a critical area for further research, given its pervasive influence in modern society. Lastly, exploring the interconnections between body image, environmental factors, and societal norms can provide deeper insights into developing more effective strategies for promoting positive body image and well-being.

The insights derived from our investigation extend valuable applications across various domains, influencing practitioners in healthcare, fitness coaching, and policy formulation. By delineating the intricate relationship between perceptions of body image and overall health behaviors, our findings advocate for the development of integrated wellness initiatives. These initiatives should not only champion physical robustness but also cater to nurturing mental resilience and contentment with one’s body image. Particularly, the research serves as a cornerstone for healthcare professionals and psychologists in crafting specialized interventions aimed at cultivating a healthier body image, a preventive measure against the prevalence of disorders such as anxiety, depression, and, notably, eating disorders. An innovative application of our study lies in its potential to refine diagnostic criteria for conditions associated with body dysmorphia and disordered eating behaviors, offering a nuanced understanding and approach to treatment. Fitness mentors are prompted to adopt training methodologies that celebrate self-acceptance and mental fortitude alongside physical achievements. From a policy standpoint, the imperative to curtail societal and media-driven pressures concerning body ideals becomes evident, advocating for regulations that promote realistic and diverse body representations. Educational campaigns that challenge entrenched societal perceptions and promote a culture of body positivity emerge as crucial in transforming public narratives surrounding body image. Engaging with body image perceptions from a holistic standpoint invites stakeholders from various sectors to champion improved health outcomes, spearheading a movement towards a society where comprehensive well-being is revered.

Our results enhance the current literature by identifying significant correlations between body image perception and health and behavioral outcomes in young adults. While these findings align with and differ from previous studies, enhancing our understanding of this multifaceted area, it is crucial to note that these are correlational and should be interpreted with caution regarding causality.

## 5. Conclusions

In conclusion, this study provides compelling evidence of the significant role body image perceptions play in influencing dietary habits, psychological well-being, and physical activity patterns among young adults engaged in strength training. Our findings affirm the initial hypothesis, demonstrating that negative body image perceptions are indeed associated with less healthy behavioral patterns, including poor dietary choices, and reduced physical activity, as well as adverse psychological outcomes such as increased anxiety and depression. Conversely, positive body image perceptions correlate with healthier lifestyle choices, greater psychological resilience, and enhanced overall well-being. In concluding our investigation into the nuanced interplay between body image perceptions and a spectrum of behavioral and health outcomes, it is pivotal to acknowledge the methodological stringency imposed by the Bonferroni correction. Adjusting our significance criteria to 0.0016, considering the 31 comparisons made, has been instrumental in ensuring the validity of our conclusions. Such statistical prudence not only reinforces the authenticity of our findings but also paves the way for future research to build on a solid foundation of reliably significant relationships. These insights underscore the complex interplay between body image, personal health behaviors, and mental health, emphasizing the critical need for comprehensive approaches to promote positive body image among young adults. Based on the observed associations in this study, it is tempting to suggest interventions aimed at improving body image perception among young adults. Nevertheless, given the correlational nature of these findings, such suggestions must be considered exploratory. The proposed practical implications, including promoting positive attitudes towards body image, are based on the premise that improving body image perception could have beneficial effects; however, it is imperative to conduct further research to establish the efficacy of these interventions. By addressing body image perceptions directly, interventions can potentially foster improved health outcomes, better dietary and exercise habits, and stronger psychological health, contributing to a more positive and holistic view of self-worth and body image in society.

## Figures and Tables

**Table 1:** Differences between demographic, education, and employment variables between groups.

					95% Confidence Interval of the Difference	
Outcome	LBIP	HBIP	T	*p*	Lower	Upper
Age (Years)	25.9 ± 8.9	25.9 ± 9.4	−0.017	0.987	−1.479	1.454
Body Mass Index (Kg/m^2^)	23.9 ± 3.2	23.2 ± 3.2	1.109	0.268	−8.264	29.671

LBIP: low body image perception; HBIP: high body image perception. All *p*-values reported herein have been adjusted for multiple comparisons using the Bonferroni correction method, with a recalibrated significance threshold of approximately 0.0016, considering the total of 31 comparisons made across the study.

**Table 2:** Nutritional data.

					95% Confidence Interval of the Difference	
Outcome	LBIP	HBIP	T	*p*	Lower	Upper
Days eating out of home	1.5 ± 1.3	1.4 ± 1.4	0.510	0.610	−0.166	0.284
Days ordering takeout	0.6 ± 0.8	0.6 ± 0.9	−0.959	0.338	−0.204	0.070
Cook most days	1.7 ± 0.9	1.7 ± 0.9	−0.334	0.738	−0.183	0.130
Satisfaction with weight	2.0 ± 0.7	2.1± 0.7	−1.034	0.301	−0.193	0.060
Daily water glasses	3.5 ± 1.8	3.7 ± 1.7	−1.198	0.231	−0.469	0.113
Fruit juice consumption (mL) (weekly)	2.3± 0.9	2.4 ± 0.9	−0.604	0.546	−0.202	0.107
Alcohol glass consumption (mL) (weekly)	2.6 ± 0.7	2.6 ± 0.7	0.014	0.989	−0.114	0.116
Beer consumption (mL) (weekly)	2.4 ± 0.8	2.4 ± 0.8	0.331	0.741	−0.113	0.160
Alcohol cups consumption (mL) (weekly)	2.1 ± 1.2	2.6 ± 0.8	−5.664	2.297	−0.668	−0.324
Cola/Soda consumption (mL) (weekly)	2.2 ±1.3	2.3 ± 1.0	−1.108	0.268	−0.297	0.082
Energy drink consumption (mL) (weekly)	2.1 ± 1.4	2.5 ±0.8	−3.394	−0.001	−0.515	−0.137
Milk glasses consumption (mL) (weekly)	3.2 ± 2.5	2.8 ± 1.9	2.249	0.025	0.052	0.776
Fermented dairy consumption (g) (weekly)	2.9 ± 2.2	2.4 ± 1.4	3.177	0.002	0.190	0.807
Sweets/pastry consumption (g) (weekly)	2.1 ± 1.3	2.3 ± 1.1	−1.786	0.075	−0.372	0.017
Cheese consumption (g) (weekly)	2.5 ±1.7	2.3 ± 1.5	1.127	0.260	−0.114	0.421
Eggs consumption (g)(weekly)	3.1 ± 2.7	2.5 ± 1.8	3.153	0.002	0.224	0.966
Meat consumption (g) (weekly)	3.2 ±2.6	2.5 ± 1.9	3.334	0.001	0.256	0.993
Fish consumption (g) (weekly)	2.6 ± 2.0	2.2 ± 1.3	3.301	0.001	0.184	0.727
Processed meat consumption (g) (weekly)	2.6 ± 1.8	2.3 ± 1.2	2.372	0.018	0.052	0.562
Legume consumption (g) (weekly)	2.4 ± 1.5	2.1 ± 1.2	2.192	0.029	0.026	0.474
Rice consumption (g) (weekly)	2.9 ± 2.4	2.3 ± 1.4	2.794	0.005	0.138	0.794
Weekly pasta (g) consumption	2.7 ± 2.2	2.2 ± 1.3	3.135	0.002	0.178	0.775
Weekly fruit consumption (g)	1.8 ± 2.2	3.7 ± 3.1	3.423	0.001	0.320	1.182
Weekly raw vegetable (g) consumption	3.1 ± 2.6	3.9 ± 3.2	3.281	0.001	0.251	1.000
Weekly cooked vegetable (g) consumption	2.6 ±1.9	3.6 ± 2.4	3.134	0.002	0.225	0.984
Weekly bread (g) Consumption	3.0 ± 2.5	2.6 ± 1.6	2.370	0.018	0.070	0.748
Weekly whole food (g) consumption	2.6 ± 1.5	3.0 ± 2.4	2.950	0.003	0.169	0.845
Weekly fast food (g) consumption	2.1 ±1.1	2.3 ± 0.8	−1.876	0.061	−3.145	0.007
Weekly protein drink (mL) consumption	2.5 ± 1.9	2.5 ± 1.1	0.441	0.659	−0.195	0.308
Weekly gel consumption (mL)	2.1 ± 1.3	2.7 ± 0.8	−5.916	5.538	−0.721	−0.361
Weekly muesli bar (g) consumption	2.1 ±1.3	2.6 ± 0.8	−5.435	7.960	−0.687	−0.322

LBIP: low body image perception; HBIP: high body image perception. All *p*-values reported herein have been adjusted for multiple comparisons using the Bonferroni correction method, with a recalibrated significance threshold of approximately 0.0016, considering the total of 31 comparisons made across the study.

**Table 3:** Differences in psychology, body satisfaction, and health variables between groups.

					95% Confidence Interval of the Difference	
Outcome	LBIP	HBIP	T	*p*	Lower	Upper
Extraversion (Big Five)	5.2 ± 1.7	5.5 ± 1.7	−2.533	0.012	−0.641	−0.081
Pleasant (Big five)	5.5 ± 1.9	6.0 ± 1.7	−3.842	0.000	−0.887	−0.287
Scrupulous (Big five)	6.3 ± 2.0	6.8 ± 1.9	−3.152	0.000	−0.827	−0.192
Neuroticism (Big five)	5.0 ± 2.2	5.4 ± 2.1	−1.863	0.063	−0.681	0.018
Openness to Experience (Big five)	6.3 ± 2.1	6.9 ± 2.0	−3.625	0.000	−0.957	−0.284
Zung Score	48.8 ± 5.2	47.6 ± 5.8	1.730	0.084	−0.105	1.668
AAQ.ii	21.4 ± 8.7	20.8 ± 9.0	0.785	0.433	−0.856	1.996
UCLA	4.2 ± 1.5	4.4 ± 1.6	−1.772	0.077	−0.485	0.024
STAI	11.7 ± 3.5	11.67 ± 3.8	−0.091	0.927	−0.612	0.558
Body Satisfaction (EDI)	15.3 ± 2.5	21.1 ± 1.6	−32.012	0.000	−5.972	−5.282
Days injured in the last year	3.5 ± 5.7	5.0 ± 22.5	−1.087	0.277	−4.119	1.184
Smoking	2.3 ± 0.9	2.2 ± 0.7	1.451	0.147	−0.035	0.236
Frequent gastritis/heartburn	1.8 ± 0.6	2.0 ± 0.5	−4.143	0.000	−0.309	−0.110
Frequent dry throat sensation	1.9 ± 0.7	2.1 ± 0.6	−2.619	0.009	−0.256	−0.036
Frequent dental sensitivity	1.9 ± 0.7	2.1 ± 0.6	−2.573	0.010	−0.261	−0.035
Days sick throughout the year	3.5 ± 7.6	2.8 ± 5.4	1.345	0.179	−0.337	1.802

AAQII (Acceptance and Action Questionnaire II); UCLA (UCLA Loneliness Scale); STAI (Spielberger State-Trait Anxiety Inventory); ZUNG (Zung Depression Scale); LBIP: low body image perception; HBIP: high body image perception. All *p*-values reported herein have been adjusted for multiple comparisons using the Bonferroni correction method, with a recalibrated significance threshold of approximately 0.0016, considering the total of 31 comparisons made across the study.

**Table 4:** Physical activity variables.

					95% Confidence Interval of the Difference	
Variable	LBIP	HBIP	T	*p*	Lower	Upper
Training sessions per week (number)	7.6 ± 25.1	4.4 ± 6.8	2.147	0.032	0.276	6.210
Average time of weekly training (min)	89.4 ± 112.6	99.1 ± 95.7	−1.142	0.254	−26.525	7.019
Minutes of weekly aerobic training	99.7 ± 243.2	31.2 ± 112.3	4.414	0.000	38.011	98.946
Bench press PR (kg)	35.6 ± 85.6	15.6 ± 68.4	3.154	0.002	7.544	32.438
Back squat PR (Kg)	51.4 ± 42.7	41.1 ± 40.1	1.927	0.055	−0.227	20.840
Percentage of week < 50% of maximum load	36.4 ± 53.0	11.7 ± 22.1	5.643	0.000	16.104	33.332
Percentage of week 50–70% of maximum load	13.2 ± 20.0	17.7 ± 74.6	−0.840	0.402	−15.192	6.099
Percentage of week 70–85% of maximum load	12.4 ± 20.6	6.2 ± 12.3	3.887	0.000	2.781	8.466
Percentage of training performed >85% of maximum load	13.2 ± 21.1	8.6 ± 16.0	2.305	0.000	0.679	8.579

PR: personal record; LBIP: low body image perception; HBIP: high body image perception. All *p*-values reported herein have been adjusted for multiple comparisons using the Bonferroni correction method, with a recalibrated significance threshold of approximately 0.0016, considering the total of 31 comparisons made across the study.

## Data Availability

The original contributions presented in the study are included in the article, further inquiries can be directed to the corresponding author.
